# Undergraduate perceptions on transitioning into E-learning for continuation of higher education during the COVID pandemic in a developing country: a cross-sectional study from Sri Lanka

**DOI:** 10.1186/s12909-022-03586-2

**Published:** 2022-07-03

**Authors:** Nirma Subashini, Lahiru Udayanga, L. H. N. De Silva, J. C. Edirisinghe, M. N. Nafla

**Affiliations:** 1 Department of Horticulture & Landscape Gardening, Faculty of Agriculture & Plantation Management, Wayamba University of Sri Lanka, Makandura, Sri Lanka; 2 Department of Biosystems Engineering, Faculty of Agriculture & Plantation Management, Wayamba University of Sri Lanka, Makandura, Sri Lanka; 3 Department of Agribusiness Management, Faculty of Agriculture & Plantation Management, Wayamba University of Sri Lanka, Makandura, Sri Lanka; 4 Institute of Indigenous Medicine, University of Colombo, Colombo, Sri Lanka

**Keywords:** Acceptance, Attitudes and Practices, E-Learning, Sri Lanka, Undergraduates

## Abstract

**Background:**

The higher education was significantly influenced by the COVID pandemic within many developing countries, including Sri Lanka, necessitating to adopt more E-Learning opportunities. Therefore, this study was conducted to characterize the perceptions of Sri Lankan undergraduates to accept E-Learning in higher education, during the COVID crisis.

**Method:**

A total of 657 randomly selected undergraduates of three state universities in Sri Lanka, were recruited as the study population. Information on Socio-demographic factors, Knowledge, Attitudes and Practices on E-Learning methods were acquired using an interviewer administered questionnaire. A Probit regression model was fitted to identify the driving factors for the willingness of undergraduates to engage in E-Learning.

**Results:**

Around, 80.2% of the respondents were females. Majority were residing in semi-urban areas (44.7%), followed by rural areas (39.9%). More than two third of the respondents were familiar with E-Learning and different Learning Management Systems (LMS) that facilitate E-Learning (68.9%). Majority of the respondents (73.7%) were using different E-Learning platforms, mostly 2 to 3 days per week (25.7%). Only around one third (36.4%) had received any formal training in using LMS or other E-Learning platforms. Smart phones (77.8%) were found to be the most preferred device used for E-Learning activities, followed by computers and laptops (21.3%). Meanwhile, LMS/MOODLE (45.4%), WhatsApp/Viber (33.0%) and Zoom (32.7%), were the most commonly used E-Learning platforms. The average acceptance of E-Learning methods was found to be 70.7% among Sri Lankan undergraduates during the COVID epidemic. Based on the Probit regression, nature of the residing locality (*P=0.009*), family income (*P=0.048*), academic field (*P<0.001*) and year (*P=0.028*), knowledge on Information and Communications Technology [ICT] (*P=0.012*), and previous training experiences on E-Learning (*P<0.001*) were found as significant drivers that influence the acceptance of E-Learning practices of the Sri Lankan undergraduates.

**Conclusion:**

Adopting E-learning into higher education sector could be recognized as a viable solution to facilitate the higher education during a crisis like COVID. However, relevant authorities in Sri Lanka should take immediate actions to empower the physical resources for E-Learning, improve the basic telecommunication infrastructure and conduct appropriate training programmes to promote E-Learning among Sri Lankan undergraduates.

## Background

With the sudden onset of COVID-19 epidemic in November 2019, many countries all over the world have been significantly influenced by the COVID epidemic, leading to a high health burden and crippling many countries in terms of health, economy, transport and education etc. [1]. The requirement of restricting the human-human interactions and maintaining of social distancing have caused significant implications on the education sector, all over the world [2]. Conventionally, education has been offered in classrooms, where students can interact directly with the teachers, respecting the physical presence [3]. Therefore, continuation of the teaching and learning activities has become a challenge to almost all the countries in the world.

Similar to many developing countries in the region, the Sri Lankan government also imposed strict lockdown, curfew and social distancing strategies to curb the COVID epidemic, with the first COVID-19 case being reported on 27^th^ of January 2020 [4, 5]. The Sri Lankan education system was predominantly relying on traditional face-to-face teaching environment, with a limited attention on E-Learning avenues. Therefore, facilitation of the education process through conventional approaches became impossible at the early phases of COVID-19. Especially, the Higher Education Institutions (HEIs) faced numerous difficulties in continuing their teaching and learning practices, under this new mode of social interplay [6]. The lockdowns imposed by Sri Lankan government, forced HEIs to rapidly adopt E-Learning technologies to facilitate distance learning. Therefore, many state and non-state universities in Sri Lanka adopted online teaching since the second quarter of 2020. However, transitioning from the conventional physical classrooms into virtual education infrastructure was challenging for Sri Lanka and many developing countries [2].

E-Learning is one of the most powerful tools that could be effectively utilized in Blended Learning (BL). The BL is a popular educational model in shifting teaching learning environment of the institutions for the next era in education, while providing new opportunities for combining traditional placed based face-to-face classroom method and online educational materials in order to enhance teaching and learning [7]. E-Learning is a wide concept, which encompasses the collaborative use of the internet and other essential learning materials, tools for teaching purposes [8]. It could be defined as the combined use of internet and multimedia technologies to ease and enhance the learning facilities. Easy Accessibility, interactivity, flexibility, and digital communication are considered as the essential characteristics of E-Learning [9]. According to Abbad *et al*. [10], E-Learning refers to the learning process that happens through electronic or digital media, while Liu and Wang [11] considers E-Learning as an internet-centred, global sharing and learning method, with enhanced flexibility of learning that is conducted in a computer-generated environment.

E-learning is simply a platform and a system, which facilitates the learning process through the internet using electronic devices. E-Learning is basically divided into two categories, namely internet-based and computer-based E-Learning. Computer-based E-Learning refers to the full use of software and hardware available either in two ways: computer-assisted-learning and computer-managed-instructions [9]. Internet-based learning is an advancement of computer-based learning, where the content available in the virtual environment is used. Learning Management Systems (LMS), such as WebCT Vista, Blackboard and MOODLE, ATUTOR, video conferencing technologies like ZOOM and Google classrooms could be recognized as most widely used arenas to supplement or enrich E-Learning opportunities, through the provision of an alternative learning environment [12]. Many developing countries in the past have underutilized E-Learning and set priorities on the traditional learning processes.

E-Learning has been incorporated into the teaching and learning systems of many developed countries, and over 80% of HEIs in these countries are equipped with E-learning facilities [3]. The adoption of E-Learning has made an impact on the design, planning, administration, and management of the learning process. By the onset of COVID pandemic, only a limited number of HEIs in developing countries were laying platforms to establish educational e-strategies [13] to promote BL. A properly implemented E-Learning system is known to enable students to receive feedback and knowledge on subjects through multiple sources and platforms, enhancing student confidence in applying learned facts to the practical situations as well [14]. Further, it can elevate the sustainability of the education sectors, while ensuring that students from various backgrounds are having access to vast educational opportunities. Realizing its true potential, the higher education sector in Sri Lanka is progressively attempting to embrace more E teaching and learning opportunities within all manner of programmes.

In 2017, the Sri Lankan government had taken initiatives towards technology-based education through Higher Education for Twenty-first Century (HETC) project. Under this project, most state universities in Sri Lanka, had implemented E-Learning platforms to perform several functionalities such as planning and scheduling of courses, teacher-student evaluation, communicating with students, etc. [15]. However, the acceptability and long-term sustainability of such E-Learning platforms were low, due to limited attention on E-Learning strategies [16, 17]. The successful implementation of E-Learning systems is influenced by a variety of factors such as technology readiness, attitudes, and perceptions of both teachers and students. Availability of appropriate hardware (computers and smartphones etc.), accessibility to internet facilities and the degree of computer literacy could be recognized as the technology readiness attributes [18]. Meanwhile, perceptions of students and teachers on E-learning, specifically on technology acceptance and individual learning styles, play a crucial role in determining the success of such systems [15]. Studies have emphasized that perceived ease, flexibility and quality of course modules, usefulness, and computer anxiety of teachers and students influence the success of E-Learning systems. Further, the ability of instructors to advance themselves to online teaching and learning platforms also plays a key role [18].

With the realization of its significance during crisis situations, Sri Lankan HEIs have also shifted towards E-Learning approaches as a key pillar in BL. A previous study conducted at the University of Colombo emphasizes that positive attitudes and ICT awareness are critical factors for implementation of successful E-Learning systems [19]. Perceptions of students and lecturers on E-Learning may differ according to the availability of resources, subject content, exposure to different electronic tools, and students’ awareness about ICT. Therefore, understanding the technology readiness and perceptions of students on E-Learning, play a critical role in promoting E-Learning in the higher education [20].

However, knowledge on the technology readiness of students and lecturers, degree of technology utilization, student readiness to accept E-Learning and the potential constraints on adoption of E-Learning strategies are limited, as detailed studies on this aspect are rare in Sri Lanka. Therefore, the current study attempted to characterize the perceptions of the undergraduate community of Sri Lanka on E-Learning and their willingness to accept E-Learning, aiming to facilitate the establishment of ideal E-Learning systems. The findings of the current study would facilitate the academic policymakers to promote E-Learning systems that drive upon the student requirements, while enabling the development of effective E-Learning platforms to serve during similar crisis situations.

## Methodology

### Selection of study population

Based on the Webometrics rankings (2019 and 2020), the government universities of Sri Lanka were classified into three categories as best, moderate and low. From each category, one state university was selected randomly, leading to a total of three government universities as the University of Kelaniya, South Eastern University of Sri Lanka and Wayamba University of Sri Lanka. Undergraduates enrolled into the above three universities were selected as the study population.

### Determination of the sample size

Sri Lankan government universities host around 130,000 to 140,000 undergraduates per year. The Lwanga and Lemeshow equation [21] was used to calculate the required sample size, as 385 undergraduates. The precision was maintained as 5% and the critical value of specified confidence level (95%) was used as 1.96, while the population proportion was set as 0.5 (50%). During the fieldwork, the sample size was increased up to 657 undergraduates. Expecting a non-responsive rate of 50%, a total of 1,000 undergraduates enrolled into different degree programmes were selected based on the concept of stratified random sampling. The selection of students was done in such a way to ensure effective representation of different academic years and disciplines. The selected students were invited to participate for the current survey through e-mail, while acquiring their informed written consent. Any student, who refused to take part in this study due to religious beliefs or an opinion that it is not worth participating, were excluded from the survey.

### Data collection

Primary data were collected using an email-based survey conducted from March to May 2020. A structured online questionnaire prepared in three local languages (Sinhala, English and Tamil) was used for primary data collection. Previous literature was referred in the development process of the questionnaire. The questionnaire consisted of 36 questions grouped into four sections related to the current status and future trends of E-Learning in higher education. The survey used various types of questions, including Likert scale, multiple-choice, and open-ended questions.

The questionnaire covered the following areas: 1. Socio-demographic information (age, gender, nature of the residence locality, monthly family income, field of study and the level of study); 2. Knowledge on E-Learning (using multiple-choice (twelve) and close-ended (four) questions focusing on the knowledge on E-Learning concepts, approaches and basic ICT knowledge); 3. Practices on E-Learning and internet surfing (using six questions that focus on the experience in E-Learning, frequency of using E-Learning platforms, availability of internet facilities, frequency and duration of internet surfing); 4 Attitudes and perceptions towards E-Learning (using eight questions covering the most preferred device and media for E-Learning, level of satisfaction on internet facilities and E-Learning, perceived constraints against E-Learning, willingness to engage in E-Learning and the preferred combination of E-Learning and conventional Learning). The prepared questionnaire was evaluated by a panel of 10 experts for content validation. In addition, it was pre-tested and validated using a preliminary sample of 30 undergraduates.

### Data interpretation and statistical analysis

All collected data were double-checked and verified on the same day for completeness and consistency, prior to entering into Microsoft Access® data sheets (version, 2013). A Probit regression model was fitted to identify the driving factors for the willingness of undergraduates to engage in E-Learning. The probit model assumes an underlying latent distribution, which cannot be observed, while the researcher observes discrete outcomes of the ‘selection’ of a particular ‘alternative’ by the respondent. Here it was assumed that utility from E-Learning has a continuous distribution [z*] and the researcher observes only discrete outcome variable [y] as,

$$\genfrac{}{}{0pt}{}{y=1\ if\ {z}^{\ast }>0}{y=0\ if\ {z}^{\ast}\le 0}$$

The probit model relates a linear combination of covariates to the latent distribution, z* as;

$${z}_i^{\ast }={x}_i^{\prime}\beta +{u}_i$$

Where, *x* is a matrix of independent covariates, *β* is a vector of coefficients to be estimated. The stochastic component of the model was denoted by *u*_*i*_. The acceptance of E-Learning was considered as the response variable in the Probit analysis, assigning the numerical value of one when the utility difference between E-Learning and classroom learning is more than zero. Value of zero will be assigned, when the utility difference between E-Learning and class room learning is less than or equal to zero. The socio-demographic factors, familiarity with E-Learning, knowledge on ICT and level of training received on E-Learning were considered as predictors. A Principal Component Analysis (PCA) coupled with Varimax rotation was used to formulate a Perception score on E-Learning. Academic disciplines such as agriculture, food science, science and technology were grouped as “Science Disciplines”, while arts & social sciences and commerce and management fields were grouped as “Non-Science Disciplines” during the analysis. All statistical analysis was done using the STATA (version 16).

## Results

### Socio-demographic characteristics

Among 657 undergraduates, 80.2% were females, while majority were residing in semi-urban areas (44.7%) followed by rural areas (39.9%) Table 1. Undergraduates belonging to the age group of 23 to 25 years of age were prominent within the sample, accounting for 58.1% as indicated in Table 1. Second year undergraduates were more abundant (30.0%), followed by third year students (26.9%). A relatively higher proportion of respondents were following the “Arts & Social sciences” stream (25.7%), while undergraduates from “Commerce & Management” and “Agricultural Sciences” fields accounted for 20.4% and 18.6%, respectively. The lowest representation was from “Biological and Physical Sciences” stream as 16.1% as indicated in Table 1. In case of monthly family income, around 56.8% families of undergraduates were receiving a total income of <25,000 Sri Lankan Rupees (LKR), followed by the 25, 001 to 26, 000 LKR income category (28.8%). It was noted that only 7.5% of the respondents were belonging to families with a total family income >75, 000 LKR.

**Table 1:** Demographic and socio-economic factors

Parameter		Total Respondents	
		n	%
Gender	Male	130	19.8
	Female	527	80.2
Age (Years)	20-22	241	36.7
	23-25	382	58.1
	>25	34	5.2
Locality	Urban	101	15.4
	Semi	294	44.7
	Rural	262	39.9
Field of Study	Agricultural and Food Sciences	122	18.6
	Arts and social sciences	169	25.7
	Commerce and Management	134	20.4
	Science	106	16.1
	Technology	126	19.2
Year of Study	First Year	156	23.7
	Second Year	197	30.0
	Third Year	177	26.9
	Fourth Year	127	19.3
Family Income	<25,000	373	56.8
	25,001 to 50,00	189	28.8
	50,001 to 75,000	46	7.0
	75,001 to 100,000	21	3.2
	100,001 to 150,000	28	4.3
	>150,000	0	0

### Knowledge on E-Learning

More than two third (72.6%) of the respondents were familiar with the concept of E-Learning and knew (68.9%) different Learning Management Systems (LMS) that facilitate E-Learning. However, only 43.5% of the respondents were familiar with online conferencing Table 2. Even though, majority (67.4%) had followed different courses on Information and Communications Technology (ICT), only around one third (36.4%) had received any formal training in using LMS or other E-Learning platforms. Furthermore, 83.4% of respondents had a moderate ICT knowledge, while only 7.6% were characterized with a low degree of ICT knowledge. Interestingly, 73.7% of the respondents had used different E-Learning platforms before, mostly at a frequency of 2 to 3 days for week (25.7 %).

**Table 2:** Knowledge of the undergraduates on E Learning

Parameter		Total Respondents	
		n	%
Familiarity with E-Learning	No	180	27.4
	Yes	477	72.6
Knowledge on LMS & MOODLE	No	204	31.1
	Yes	453	68.9
Familiar with Online Conferencing	No	371	56.5
	Yes	286	43.5
Followed ICT Courses	No	214	32.6
	Yes	443	67.4
Received a formal training on E-Learning	No	418	63.6
	Yes	239	36.4
Knowledge on ICT	Low	50	7.6
	Moderate	548	83.4
	High	59	9.0

*Note: ICT* Information and Communications Technology*, LMS* Learning Management System*, MOODLE* An Open-source Learning Platform

### Perceptions on E-Learning

Majority of the respondents claimed to prefer smart phones (77.8%) for E-Learning activities, while tabs were least preferred (0.9%). In case of the most widely used E-Learning platform, LMS/MOODLE (45.4%), WhatsApp/Viber (33.0%) and Zoom (32.7%), outcompeted the rest Table 3. A relatively higher proportion of undergraduates (43.7%) were satisfied with the internet facilities provided for E-Learning Practices, while less than 10% remained dissatisfied. Even though, more than three quarter of respondents (77.6%) were willing to recommend E-Learning methods to their colleagues, only 70.9% students were willing to use E-Learning methods by themselves.

**Table 3:** Perceptions of the undergraduates on E Learning

Parameter		Total Respondents	
		n	%
Most Preferred Device for E-Learning	Smart Phone	511	77.8
	Tab	6	0.9
	Lap/Computer	140	21.3
Most preferred E Learning Media	Computer aided Learning (CAL)	1	.2
	E-mails	78	11.9
	Facebook	7	1.1
	Internet	2	.3
	LMS/MOODLE	298	45.4
	Virtual Learning Environment (VLE)	2	0.4
	WhatsApp/Viber	217	33.0
	Zoom	215	32.7
Satisfaction on Internet facilities at the University	Highly Dissatisfied	19	2.9
	Dissatisfied	50	7.6
	Neutral	199	30.3
	Satisfied	287	43.7
	Highly Satisfied	102	15.5
Constrictions in E-Learning	Limited internet access	511	77.8
	Poor signals in mobile networks at home	528	80.4
	Poor understanding of the E-Learning systems	220	33.5
	Lecturers/instructors do not provide clear instructions for using e-contents	139	21.2
	Language difficulties	188	28.6
	LMS is not updated properly	189	28.8
	Lack of physical help from the lecturers	218	33.2
	Less motivation due to lacking face to face interactions	248	37.7
	Less evaluation in learning outcomes	221	33.6
Willingness for E Learning	Yes	466	70.9
	No	191	29.1
Recommended E Learning	Yes	510	77.6
	No	147	22.4
Willingness for E-Learning at Crisis situations	Yes	512	77.9
	No	145	22.1
Most Preferred E-Learning Combination	100% Traditional	40	6.1
	75% Traditional +25% E Learning	250	38.1
	50% Traditional +50% E Learning	302	46.0
	75% Traditional +25% E Learning	45	6.8
	100% E Learning	20	3.0

*Note: CAL* Computer aided Learning*, ICT* Information and Communications Technology, *LMS* Learning Management System*, MOODLE* An Open-source Learning Platform*, VLE* Virtual Learning Environment

Poor signals in mobile networks at home (80.4%), limited internet access (77.8%), less motivation due to lack of face-to-face interactions (37.7%), lack of physical help from the lecturers (33.2%) and poor understanding of the E-Learning systems (33.5%) were recognized as the major constraints faced by the undergraduates, when using E-Learning techniques. However, 77.9% of undergraduates were willing to shift towards E-Learning methods during a crisis such as COVID 19. Nearly half of the respondents (46.0%) were preferring 50% Traditional +50% E Learning combination to continue their studies, while 100% E-Learning methods was least preferred (3.0%), as indicated in Table 3.

### E-Learning practices of undergraduates

Majority of respondents (73.7%) had already used different E-Learning platforms before, mainly when instructed by the lecturer (25.9%) or two-three times per week (25.7%) as indicated in Table 4. It was noted that approximately, two third of undergraduates (66.7%) had internet facilities at home. In general, 50.4% of respondents were spending 1 -2 hours for internet surfing per day, followed by 2- 3 hours (32.4%). However, it was noted that a notable fraction of undergraduates (11.6%) were spending less than 30 minutes for internet surfing per day, even free internet facilities are provided at the university Table 4.

**Table 4:** Practices of the undergraduates on E Learning

Parameter		Total Respondents	
		n	%
Previous experience in E-Learning	No	173	26.3
	Yes	484	73.7
How often	Daily	126	19.2
	2-3 days per week	169	25.7
	Weekly	95	14.5
	Monthly	97	14.8
	When instructed by Lecturer	170	25.9
Having Permanent Internet Facility at Home	No	219	33.3
	Yes	438	66.7
Internet Surfing Time (Hours)	< 0.5	76	11.6
	0.5-1	19	2.9
	1-2	331	50.4
	2-3	213	32.4
	>4	18	2.7

### Driving factors of E-Learning

The results of the Probit model denoted that, only the residing location (*P=0.009*), family income (*P=0.048*), academic field (*P<0.001*) and year (*P=0.028*), knowledge on ICT (*P=0.012*) and previous training experiences on E-Learning (*P<0.001*) showed a significant influence on the acceptance of E-Learning techniques (Table 5) at a 95% confidence level. In addition, the ‘Internet Readiness’ remained significant (*P=0.061*) at a significance level of 10%. Availability of a satisfactory internet connection and an appropriate physical devise to engage in E-Learning or surf in internet was considered as “Internet Readiness”. Meanwhile, gender and familiarity (*P=0.748*) with E-Learning platforms (*P=0.248*) remained non-significant Table 5.

**Table 5:** Outcomes of the Probit Model

Parameter	Coefficient	Overall P value	95% Confidence Interval	
			Lower	Upper
Constant	1.428	<0.001	0.739	2.117
Residing Locality				
*Semi Urban*	-0.394	0.009	-0.775	-0.012
*Rural*	-0.575		-0.959	-0.191
Gender				
*Male*	0.048	0.748	-0.246	0.342
Familiarity with E-learning				
*Yes*	-0.154	0.298	-0.442	0.135
Previous LMS Training				
*Yes*	1.079	<0.001	0.783	1.375
ICT Knowledge				
*Moderate*	0.162	0.012	-0.295	0.620
*High*	0.953		0.261	1.645
Family income (LKR)				
*25,001 to 50,00*	0.058	0.048	-0.204	0.319
*50,001 to 75,000*	0.421		-0.068	0.910
*75,001 to 100,000*	0.586		-0.393	1.565
*100,001 to 150,000*	1.329		0.263	2.395
Internet readiness	0.022	0.061	-0.001	0.044
Academic Year				
*Second Year*	-0.541	0.028	-1.048	-0.033
*Third Year*	-0.595		-1.141	-0.049
*Fourth Year*	-0.898		-1.531	-0.264
Field of study				
*Science Disciplines*	-1.310	<0.001	-1.817	-0.802

*Note: ICT* Information and Communications Technology*, LMS* Learning Management System

According to the predicted marginal values shown in the Table 6, undergraduates residing in urban areas had a significantly higher acceptance level of E-Learning (80.3%), compared to undergraduates from semi-urban (71.4%) or rural areas (66.7%). In case of gender, both male (71.6%) and female (70.5%) students denoted more or less similar acceptance levels. Interestingly, undergraduates with no familiarity with E-Learning systems showed a relatively higher willingness towards E-Learning (73.5%), than those with previous experience (69.7%) Table 6.

**Table 6:** Predicted marginal values of the Probit Model

Parameter	Margin	Std. Err	95% Confidence Interval	
			Lower	Upper
Residing Locality				
*Urban*	0.803	0.035	0.734	0.872
*Semi Urban*	0.714	0.022	0.670	0.758
*Rural*	0.667	0.024	0.620	0.715
Gender				
*Female*	0.705	0.017	0.673	0.737
*Male*	0.717	0.032	0.654	0.780
Familiarity with E-learning				
*No*	0.734	0.029	0.677	0.791
*Yes*	0.697	0.018	0.662	0.732
Previous LMS Training				
*No*	0.601	0.021	0.561	0.641
*Yes*	0.850	0.019	0.813	0.888
ICT Knowledge				
*Low*	0.657	0.061	0.537	0.776
*Moderate*	0.699	0.016	0.668	0.731
*High*	0.865	0.043	0.780	0.950
Family income (LKR)				
*<25,000*	0.684	0.020	0.644	0.724
*25,001 to 50,00*	0.699	0.028	0.645	0.753
*50,001 to 75,000*	0.785	0.051	0.685	0.885
*75,001 to 100,000*	0.819	0.096	0.631	1.006
*100,001 to 150,000*	0.927	0.054	0.821	1.034

*Note: ICT* Information and Communications Technology*, LMS* Learning Management System

However, undergraduates with higher ICT knowledge were characterized with significantly higher acceptance level (86.5%) of E-Learning techniques, in comparison to undergraduates with moderate (69.9%) and poor (65.7%) ICT knowledge. The acceptance of E-Learning gradually increased with the elevating family income level. Respondents with a total family income of 100,001 to 150, 000 LKR showed the highest acceptance level of E-Learning as 92.7%, while the lowest acceptance level (68.3%) was observed from families with income <25,000 LKR Table 6.

**Fig. 1:**
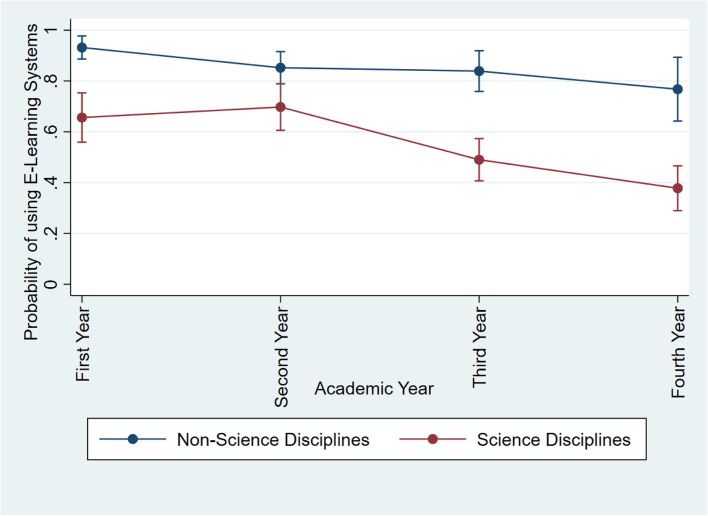
Probability of accepting E-Learning methods by undergraduates from “Science” and “Non-Science” disciplines

In case of the academic field, undergraduates from “Science Disciplines” were having a relatively lower acceptance level on E-Learning, when compared to the respondents from “Non-science Disciplines” Tables 5 and 6. The academic year of respondents indicated a negative association with the willingness towards E-Learning, suggesting that undergraduates prefer less E-Learning involvement at higher academic years. In the undergraduates of “Non-Science Discipline”, the highest acceptance level was observed from first year students from as 93.3%, while the lowest was observed as 77.2% among fourth year undergraduates (Fig. 1). On the contrary, the highest acceptance level among undergraduates from “Science Discipline” remained lower (65.9%) than the lowest acceptance of “Non-Science” students. This trend was apparent in undergraduates from both “Science” and “Non-Science” disciplines, except for second year students of “Science Discipline”. However, the gradual decay in E-Learning acceptance was relatively higher in undergraduates from “Science Discipline” (Fig. 1). Based on the predictions of the Probit model, the average acceptance of E-Learning techniques by a Sri Lankan undergraduate was found to be 70.7% (67.9% to 73.6% as 95% confidence levels).

## Discussion

E-Learning is an important aspect in transforming the conventional academic interactions in to a more technologically facilitated process, which bear many benefits to both learners and teachers. Lifelong learning and flexibility play an increasingly important role in higher education, where many studies have indicated the increasing role of technologies in higher education to improve the education experiences by enhancing methods of mobile teaching/learning [22]. The e-platforms are highly useful, when a lecturer is not physically available and at instances like COVID-19 epidemics, where social distancing is essential. Further, this may facilitate learning with busy schedules, especially for older students with occupational involvements [23]. At present, it is evident that blended learning approaches are being adopted in higher education system in a significant way.

### Knowledge on E-learning

Technology in the educational process is not new, and certain E-Learning applications have become more pervasive. Therefore, it is necessary to understand the knowledge level on E-Learning applications with the impact of technology used in learning options. According to the findings, approximately two third of the study population was familiar with the E-Learning concepts and LMS. Nevertheless, around 44% of the studied group was not familiar with online conferencing. According to Kim & Bonk [23], video conferencing technology would significantly enhance the delivery of online learning, especially with field experts. Since, around one third of study population had received formal trainings in using E-Learning platforms, Sri Lankan universities have to pay more attention on training the students on different E-Learning platforms. Another recent study has also emphasized that universities have to provide support to enhance students’ self-efficacy of using LMS, which could lead to higher student learning outcomes [24].

### Perceptions on E-Learning

The level of access to E-Learning and associated technologies was generally high across the study population, where most of the students were either owning or having access to smart phones, laptop computers or desktop computers to engage in E-Learning. The undergraduate population in the current study reported a higher preference in utilizing smart phones in their learning activities (77.8%). This finding is aligning with few recent studies, where smart phone has been reported as the most commonly used device, followed by notebook computers for online learning activities [25]. Limitations in family income, ability of multi-tasking and easy handling could be the major divers for the preference of smart phones by students.

In case of online platforms, several studies have shown that students are very familiar with social media, web-video (YouTube), and chat options like WhatsApp and Viber. Interestingly, LMS/MOODLE, WhatsApp/Viber and Zoom turned-out to be the most prominent E-Learning platforms in the current study. This finding is supported by several studies conducted in developing countries [12], where social media sites have been found to be the most frequently used platform for networking by the students, while online learning systems at universities (E-Learning Space/LMS) have emerged as the most prominently used platforms for education [26, 27]. However, at present Zoom is the most widely used E-Learning and conferencing platform during the COVID pandemic, due to the provision of free accounts for the University academics with data free options. E-Learning systems can be combined with different analytical approaches and more attractive web-based learning models, to enhance the teaching and learning activities. This will ensure provision of a BL experience to the undergraduates [7, 16]. Around 70.9% of respondents were willing to use E-Learning methods during their higher education. Many previous studies conducted to assess the student satisfaction in online courses or programmes, have reported both satisfied and dissatisfied students with E-Learning [23].

The current study disclosed that a relatively higher proportion of undergraduates (43.7%) were satisfied with the facilities provided by the University for E-Learning, while around 77.6% were willing to recommend E-Learning methods to their colleagues. This suggested that students tend to accept E-Learning sensibly. However, two previous studies, have also found that the satisfaction and acceptance of E-Learning was above average [28, 29]. According to Rogers [30], once a person starts using and becomes familiar with an E-Learning system, they may begin to persuade their colleagues and friends to adopt it, promoting such E-Learning platforms. Therefore, educators can promote E-Learning among potential early adopters, who tend to have a higher level of personal expertise in IT to popularize.

### E-Learning ZRACTICES of Undergraduates

Majority of the students were using E-Learning platforms as per the instruction by the lecturer, where only 25.7% of the students were using E-Learning facilities 2-3 times per week. In case of internet surfing time, 1-2 hours per day was the most frequent duration, followed by 2-3 hours. A recent study has revealed that the degree of internet surfing by males is higher than that of females, where majority spend ≥ 3 hours for internet surfing per day in China [31]. Another study, has reported that majority of the respondents spend 3-4 hours for E-Learning, while only 4% of the respondents spend over 6 hours on a daily basis [14]. Spending more surfing time in internet may increase the technical familiarity of the students, thereby enhancing the acceptance of E-Learning methods.

### Driving factors for E-Learning

A variety of student characteristics such as gender, age, previous knowledge on ICT, attitudes toward new technologies and learning style may result a powerful influence on E-Learning practices of students [12]. Findings of the current study report that residing location, family income, academic field and year, knowledge on ICT, and previous training experiences on E-Learning are significantly contributing to shape up the E-Learning practices of the Sri Lankan undergraduates. Several recent studies have reported that gender doesn’t denote any significant influence on the success and usage of E-Learning platforms, which is in agreement with the findings of this study [12, 22]. The acceptance of E-Learning was significantly associated with the ICT knowledge, where undergraduates with higher ICT literacy were characterized with significantly higher acceptance level for E-Learning (86.5%). Many studies have supported the above claim, while findings of Naveh *et al.* [32] have revealed that computer literacy does not enhance student acceptance or satisfaction on E-Learning environment [33, 34].

The results denoted a strong correlation between the academic fields of study with the acceptance level of E-Learning, where undergraduates from “Science disciplines” showed a relatively lower acceptance level on E-Learning, compared to the respondents from “Non-science Disciplines”. Undergraduates following science related disciplines undergo different laboratory or field based practical sessions, which develop a core skill set relevant to application, analysis, evaluation and synthesis of knowledge. Difficulties faced in covering such practical activities via E-Learning platforms and inability of acquiring hands-on experience may be the reasons behind this observation. However, a previous study by Naveh *et al.* [32], has suggested that the relationship of course discipline with usage of E-Learning platforms is rather weak. According to his findings, students following exact sciences are slightly less satisfied with course websites, compared to their counterparts in non-exact sciences, despite that the majority (53%) of the online available courses belonged to the exact science category (health, engineering and natural faculties). On the contrary, Smith *et al.* [35] has mentioned that students from mathematics and natural-science disciplines use LMS more often than students following social sciences and humanity courses.

Interestingly, a negative association was observed among the academic year of respondents and the willingness towards E-Learning, suggesting that undergraduates in final years show less preference for E-Learning. Findings of Trow [36] has evidenced that LMS use and satisfaction among first-year students were relatively higher, while Harasim [37] has suggested that E-Learning platforms serve as question and answering platforms for first year undergraduates, enabling them to update their theoretical knowledge easily. However, with time, undergraduates in higher academic years prefer more practical exposure and hands-on experience rather than theoretical knowledge, which remains difficult to be catered through E-Learning platforms. In addition, being accustomed to in-person teaching and learning methods could also be a potential reason behind the higher resistance towards shifting into E-learning by undergraduates in higher academic years. Therefore, around half of the respondents were preferring a combination of traditional and E-Learning (50:50), while 100% E-Learning approach was least preferred.

### Way forward & recommendations

Even though E-Learning is considered as an effective way of delivering educational facts, it has to be carefully designed considering the emotional and social needs of students. Further, E-Learning systems have to cater for individual requirements such as the degree of self-motivation, self-management, self-control, and time management of students [38]. Therefore, developing the core skills and competencies among the students and the provision of essential physical resources (computers & networking) is vital in obtaining a better outcome through E-Learning.

Sri Lankan instructors show a positive and supportive attitude on E-Learning initiatives. Even with better attractive, feasible, and efficient E-Learning practices, instructors prefer a mixed learning environment. Poor internet connection, insufficient server capacities, poor maintenance of computers have been identified as major issues to be dealt with in E-Learning platforms in the above study [39]. From the student perspective, teacher and facilitators’ view, teaching and learning activities, student support, access, flexibility, attitudes on E-Learning, students’ academic confidence and localization of content have been identified as seven major challenges for promotion of E-Learning in a developing country like Sri Lanka [40]. Meanwhile, Vidanagama [41] has revealed that building up positive attitudes towards E-Learning and developing IT infrastructure and related technologies could increase the acceptance of E-Learning platforms by the undergraduates.

Critics of online education have questioned the value, effectiveness, and quality of the E-Learning systems. According to Bauk *et al.* [7], the absence of proper audio/video instructional materials and the inability to provide a better user-friendly environment may lead to customer dissatisfaction. In addition, the absence of E-Learning system stability/reliability could cause a greater customer dissatisfaction. Although online learning systems are considered as a viable alternative to the traditional in-class learning activities, hesitancy of academic members to support online platform could lead to problems in distance learning. This fact is additionally supported by Nelson & Thompson [42], which implies lack of administrative support, workload, and equipment concerns as linking issues. Similarly, lack of technical expertise, slow internet connections, and unavailability of proper hardware and software resources are also considered as limiting factors for teaching via E-Learning systems [43].

The current study was conducted during the first wave of COVID 19, when E-learning began to get promoted in the local universities. At present, E-Learning via LMS, ZOOM and Google Team has become the predominant method for teaching and learning activities in Sri Lanka. A recall bias could have occurred in the current study, which remains as a general limitation in this type of studies.

## Conclusions

In conclusion, findings of the current study reveal that a higher fraction of the undergraduates are willing to use E-Learning platforms during their learning process. Further, 50% Traditional +50% E Learning combination was the most preferred combination of blended learning. On the contrary, 100% E-Learning was the least preferred approach. Students from “Science Disciplines” were relatively reluctant to shift into E-Learning methods, when compared to “Non-science Disciplines”. Further, undergraduates in later academic years denoted a lower affinity towards E-Learning. Nature of the residing locality, family income, academic field and year, knowledge on ICT, and previous training experiences on E-Learning were found as significant drivers that influence the acceptance of E-Learning practices of the Sri Lankan undergraduates. The average acceptance of E-Learning methods was found to be 70.7% (67.9% to 73.6%) among Sri Lankan undergraduates. Therefore, the University Grants Commission and Higher Education Ministry in Sri Lanka should pay more attention towards these factors and reported limitations to promote E-Learning approaches in Sri Lanka, while ensuring BL. Therefore, empowering the physical resources for E-Learning, improving the basic telecommunication and conducting appropriate training programmes on E-Learning methods, are recommended to promote E-Learning among Sri Lankan undergraduates. In addition, further in-depth studies are recommended to be conducted to identify the underlying reasons behind the constraints for E-Learning and to develop viable solutions.

## Data Availability

Data supporting the conclusions of this article are included within the article. Further data can be acquired through a proper request from the corresponding author.

## Abbreviations

BL: Blended Learning
CAL: Computer aided Learning
HEI: Higher Education Institutions
HETC: Higher Education for Twenty-first Century
ICT: Information Communication Technology
LMS: Learning Management System
PCA: Principal Component Analysis
VLE: Virtual Learning Environment
WHO: World Health Organization
